# Improved Production of 2,3-Butanediol in *Bacillus amyloliquefaciens* by Over-Expression of Glyceraldehyde-3-Phosphate Dehydrogenase and 2,3-butanediol Dehydrogenase

**DOI:** 10.1371/journal.pone.0076149

**Published:** 2013-10-02

**Authors:** Taowei Yang, Zhiming Rao, Xian Zhang, Meijuan Xu, Zhenghong Xu, Shang-Tian Yang

**Affiliations:** 1 The Key Laboratory of Industrial Biotechnology, Ministry of Education, School of Biotechnology, Jiangnan University, Wuxi, Jiangsu Province, China; 2 Laboratory of Pharmaceutical Engineering, School of Medicine and Pharmaceutics, Jiangnan University, Wuxi, Jiangsu Province, China; 3 Department of Chemical and Biomolecular Engineering, the Ohio State University, Columbus, Ohio, United States of America; University of Texas Health Science Center at San Antonio, United States of America

## Abstract

**Background:**

Previously, a safe strain, *Bacillus amyloliquefaciens* B10-127 was identified as an excellent candidate for industrial-scale microbial fermentation of 2,3-butanediol (2,3-BD). However, *B. amyloliquefaciens* fermentation yields large quantities of acetoin, lactate and succinate as by-products, and the 2,3-BD yield remains prohibitively low for commercial production.

**Methodology/Principal Findings:**

In the 2,3-butanediol metabolic pathway, glyceraldehyde-3-phosphate dehydrogenase (GAPDH) catalyzes the conversion of 3-phosphate glyceraldehyde to 1,3-bisphosphoglycerate, with concomitant reduction of NAD^+^ to NADH. In the same pathway, 2,3-BD dehydrogenase (BDH) catalyzes the conversion of acetoin to 2,3-BD with concomitant oxidation of NADH to NAD^+^. In this study, to improve 2,3-BD production, we first over-produced NAD^+^-dependent GAPDH and NADH-dependent BDH in *B. amyloliquefaciens*. Excess GAPDH reduced the fermentation time, increased the 2,3-BD yield by 12.7%, and decreased the acetoin titer by 44.3%. However, the process also enhanced lactate and succinate production. Excess BDH increased the 2,3-BD yield by 16.6% while decreasing acetoin, lactate and succinate production, but prolonged the fermentation time. When BDH and GAPDH were co-overproduced in *B. amyloliquefaciens*, the fermentation time was reduced. Furthermore, in the NADH-dependent pathways, the molar yield of 2,3-BD was increased by 22.7%, while those of acetoin, lactate and succinate were reduced by 80.8%, 33.3% and 39.5%, relative to the parent strain. In fed-batch fermentations, the 2,3-BD concentration was maximized at 132.9 g/l after 45 h, with a productivity of 2.95 g/l·h.

**Conclusions/Significance:**

Co-overexpression of *bdh* and *gapA* genes proved an effective method for enhancing 2,3-BD production and inhibiting the accumulation of unwanted by-products (acetoin, lactate and succinate). To our knowledge, we have attained the highest 2,3-BD fermentation yield thus far reported for safe microorganisms.

## Introduction

2,3-butanediol (2,3-BD) is a colorless, odorless liquid adopted in numerous industrial applications. 2,3-BD is a potentially valuable fuel additive with a heating value of 27.2 kJ g^-1^, comparable to that of other liquid fuels (e.g. methanol 22.081 kJ g^-l^, ethanol 29.055 kJ g^-l^) [1]. 2,3-BD can be dehydrated to yield methyl-ethyl ketone (an industrial solvent) [2] or 2,3-butadiene (an important precursor of synthetic rubber) [3]. Because they are more ecologically friendly and economical than traditional chemical methods, 2,3-BD production biotechnologies have attracted growing attention in recent years [4,5].

Various strains such as *Bacillus polymyxa* [6], *Serratia marcescens* [7], *Klebsiella oxytoca* [8] and *K. pneumoniae* [9] produce high 2,3-BD titers from a broad spectrum of substrates. However, the pathogenic nature of these organisms precludes their use in industrial-scale 2,3-BD fermentation [5,10]. By contrast, the Gram-positive bacterium *B. amyloliquefaciens* has been classified as GRAS (generally regarded as safe) by the US Food and Drug Administration [11]. Previously, we isolated a safe strain, *B. amyloliquefaciens* B10-127, that produces 2,3-BD from glucose, and demonstrated the potential of the strain for industrial-scale 2,3-BD production [12,13]. However, this strain also generates large quantities of unwanted by-products (acetoin, lactate and succinate), and insufficient 2,3-BD yield for commercial production. Therefore, efforts to increase 2,3-BD production by *B. amyloliquefaciens* are economically important.

**Figure 1:**
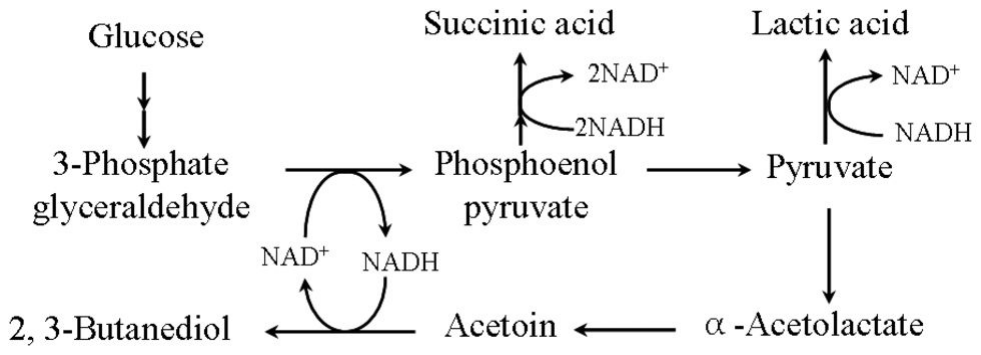
Mixed acid-2,3-BD pathway.

The 2,3-BD pathway has been studied in a range of bacteria [14]. As shown in Figure 1, assimilation of glucose to produce 2,3-BD is also an oxido-reduction-associated process. In the oxidative pathway, glucose is converted to 3-phosphate glyceraldehyde. The NAD^+^-dependent glyceraldehyde-3-phosphate dehydrogenase (GAPDH) oxidizes 3-phosphate glyceraldehyde to 1,3-bisphosphoglycerate, which is subsequently oxidized to pyruvate. Acetolactate synthase catalyzes the in vivo coupling of two pyruvate molecules to form acetolactate, which is then decarboxylated to acetoin by acetolactate decarboxylase [15]. Finally, acetoin is reduced to 2,3-BD by an NADH-dependent 2,3-BD dehydrogenase (BDH) [16,17]. In the alternative NADH-dependent pathways, succinate and lactate are co-produced from pyruvate. Bacterial strains may accumulate acetoin for several reasons. One factor that limits acetoin degradation is low levels of BDH, assumed as the rate-limiting factor in the conversion of acetoin into 2,3-BD. Alternatively, low levels of NADH may limit the BDH reaction, since this coenzyme is preferentially used in 2,3-BD synthesis. In this paper, we report over-production of NAD^+^-dependent GAPDH and NADH-dependent BDH in *B. amyloliquefaciens*, and its consequential effects on glucose metabolism.

## Materials and Methods

### Strains and plasmids construction

Strains, plasmids and primers used in this study are listed in Table 1. The parent strain was *B. amyloliquefaciens* B10-127. This strain has been deposited in the China Center for Type Culture Collection (CCTCC) under collection number CCTCC M 2012349. The recombinant derivatives of *E. coli* / *B. subtilis* shuttle plasmid vector pMA5-HapII were hosted in *Escherichia coli* JM109. This shuttle vector introduced the expression cassette for hyper-expression of *bdh* and *gapA* into *B. amyloliquefaciens* B10-127.

**Table 1:** Strains, plasmids and primers used in this study.

**Bacterial strain, plasmid or primer names**	**Relevant characteristic or sequence**	**Source or enzyme site**
**Strains**		
*B. amyloliquefaciens*		
B10-127		Laboratory stock
pBDH	B10-127 with pMA5-*bdh*	This study
pGAP	B10-127 with pMA5-*gapA*	This study
pBG	B10-127 with pMA5-*bdh-*HapII-*gapA*	This study
*E. coli* JM109		Laboratory stock
**Plasmids**		
pMA5-HapII	Expression vector (in *E. Coli*, Ap^r^; in *B. amyloliquefaciens*, Kan^r^)	Laboratory stock
pMA5-*bdh*	pMA5-HapII with *bdh* (in *E. Coli*, Ap^r^; in *B. amyloliquefaciens*, Kan^r^)	This study
pMA5-*gapA*	pMA5-HapII with *gapA* (in *E. Coli*, Ap^r^; in *B. amyloliquefaciens*, Kan^r^)	This study
pMA5-*bdh*- HapII-*gapA*	Ap^r^, Kan^r^; pMA5-HapII with *bdh* and HapII-*gapA* (in *E. Coli*, Ap^r^; in *B. amyloliquefaciens*, Kan^r^)	This study
**Primers**		
P1	5′-CATATGATGAAAGCGGCAAGATGGC-3′	*Nde*I
P2	5′-GGATCCTTAATTCGGTTTTACTAAG-3′	*Bam*HI
P3	5′-GGATCCATGGCAGTAAAAGTCGG-3′	*Bam*HI
P4	5′-ACGCGTTTAAAGACCTTGTTTTGCG-3′	*Mlu*I
P5	5′-ACGCGT TTTTGAGTGATCTTCTC-3′	*Mlu*I

Underlined nucleotides are the restriction enzyme sites

**Figure 2:**
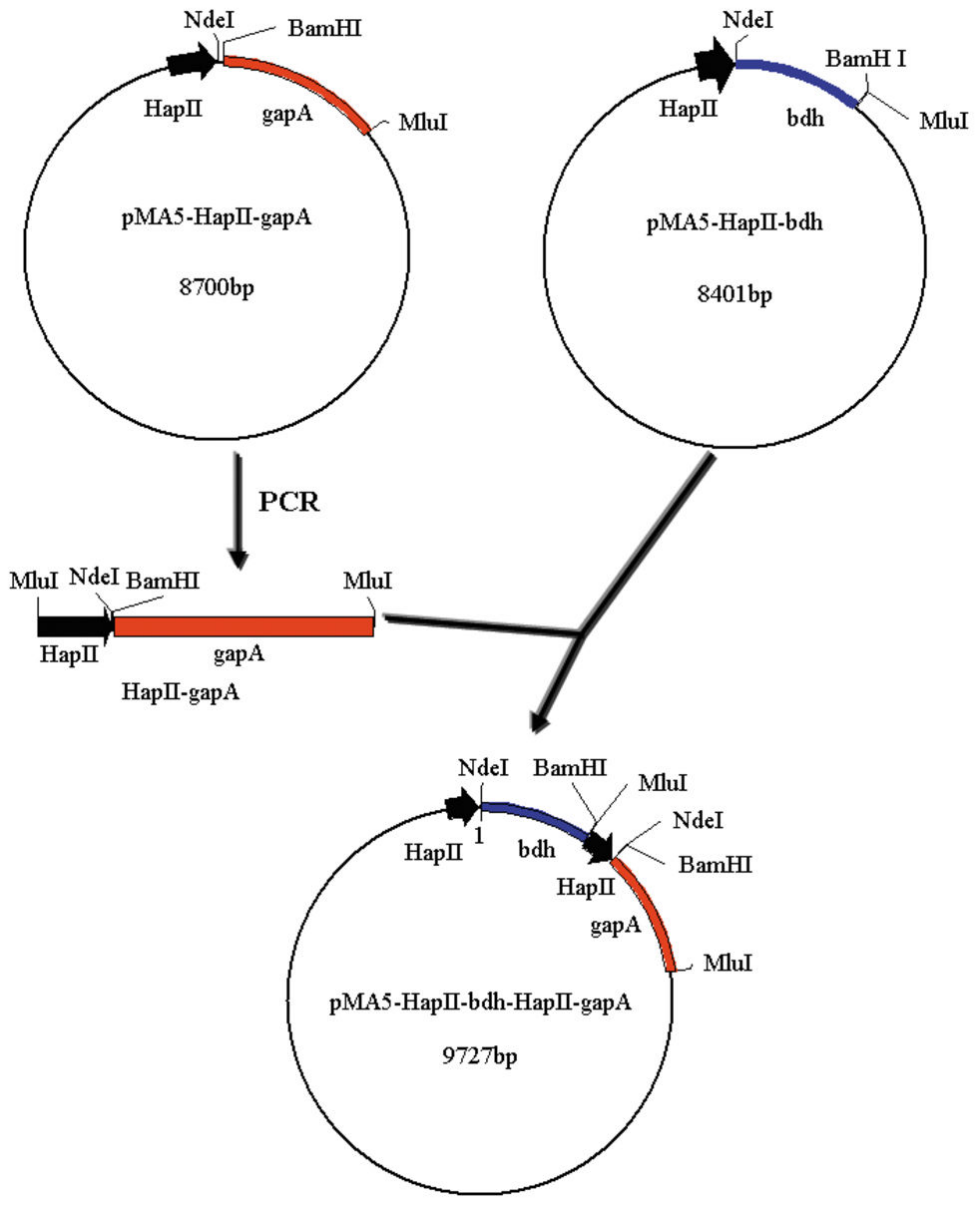
The construction of pBG.

The *bdh* gene encoding 2,3-BD dehydrogenase (BDH) from *B. amyloliquefaciens* was amplified by PCR using primers P1 and P2 (see Table 1). The *gapA* gene encoding glyceraldehyde-3-phosphate (GAPDH) from *B. amyloliquefaciens* was PCR-amplified using primers P3 and P4. The amplified *bdh* gene was inserted into the *Nde*I and *Bam*HI sites of the pMA5-HapII plasmid to create the pMA5- *bdh* plasmid. The amplified *gapA* gene was inserted into the *Bam*HI and *Mlu*I sites of the pMA5-HapII plasmid to create the pMA5-*gapA* plasmid. The *gapA* gene, containing the HapII promoter from the pMA5-*gapA* plasmid, was then PCR-amplified using primers P4 and P5. The amplified HapII-*gapA* gene was inserted into the *Mlu*I site of the pMA5-*bdh* plasmid to create the pMA5-*bdh*-HapII-*gapA* plasmid (Figure 2). The plasmids were transformed into *E. coli* JM109 cells by the calcium chloride method [18]. These constructed plasmids were subsequently isolated from *E. coli* JM109 and transformed into *B. amyloliquefaciens*, using the method described by Vojcic et al. [19].

The stability of plasmids in *B. amyloliquefaciens* has been previously tested [20]. Appropriately diluted fermentation samples were spread onto selective (50 µg/mL kanamycin) and nonselective LB agar plates. The plates were incubated at 37 °C for 14-18 h. Plasmid stability was determined as the ratio of number of colonies on the antibiotic agar plates to the number on the non-selective plates.

### Media and culture conditions

Nutrient broth, containing 40 g/l glucose, 10 g/l peptone, 5 g/l yeast extract, and 10 g/l NaCl, was used as the pre-culture medium. The medium was sterilized at 121 °C for 20 min. The 2,3-BD production medium (pH = 6.5) comprised 200 g/l glucose, 30 g/l corn steep liquor, 20 g/l soybean meal, 5 g/l ammonium citrate, 0.6 g/l succinic acid, 3 g/l K _2_HPO_4_·12H_2_O, 0.3 g/l MgSO_4_·7H_2_O.

The seed culture was agitated in 250-ml shake flasks for 10 h (160 rpm in a reciprocal shaker) at 37 °C. For batch fermentation, 2.5 l of fermentation medium was inoculated with 150 ml seed culture in a 5-l fermenter (BIOTECH-2002, Baoxing Biological Equipment Co., Shanghai, China). The agitation speed and aeration rate were 350 rpm and 0.33 vvm, respectively.

### Analysis of cell growth and metabolites

The cell mass concentration was determined from the OD at 600 nm in a UV-visible spectrophotometer (UV-2000, UNICO). The dry cell weight (DCW) was calculated from the optical density measurement, using a calibration curve for the strain.

The composition of the fermentation broth (glucose, 2,3-BD, acetoin, acetate and succinate) was determined by high-performance liquid chromatography (HPLC) [13].

### Determination of NAD^+^ and NADH concentrations

The intracellular concentrations of NAD^+^ and NADH were determined as described in previous studies [21,22].

### Assay of 2,3-BD dehydrogenase and glyceraldehyde-3-phosphate dehydrogenase activity

Assays of 2,3-BD dehydrogenase and its activity were prepared as previously described [23,24].

Ferdinand et al. [25] reported that A_340_ measurements are increased by cell extracts. From the resulting linear increase in A_340_, the glyceraldehyde-3-phosphate dehydrogenase activity can be measured.

## Results

### Over-expression of *gapA* gene and its effects on glucose fermentation to 2,3-BD in *B. amyloliquefaciens*

Glyceraldehyde-3-phosphate dehydrogenase (GAPDH, encoded by *gapA*), catalyzes the conversion of 3-phosphate glyceraldehyde to 1,3-bisphosphoglycerate, with concomitant reduction of NAD^+^ to NADH. In this study, GAPDH was cloned from *B. amyloliquefaciens* B10-127 and over-expressed in the pGAP strain, which harbored the pMA5-*gapA* plasmid. The plasmid stability was measured as described in Materials and Methods. The plasmid genetic rate remained about 96%, suggesting that pMA5-*gapA* was stably expressed in the pGAP. As shown in Figure S1, GAPDH was successfully over-expressed in the pGAP strain. Moreover, time profiles of the specific activities of GAPDH was observed during the 2,3-BD fermentation (Figure S2A). At 12^th^ h, the specific activity of GAPDH was about 1.19-fold higher than in the wild type strain. And at 24^th^ h, it was about 3.14-fold higher than in the parental strain. And then their activities reached the stationary phase. Thus, the enzyme was successfully over-expressed and its performance improved by increasing the gene dosage.

In both the parent and the engineered strains, intracellular concentrations of NADH and NAD^+^ continuously changed during various stages of the batch fermentation (Figure S3). Furthermore, over-production of GAPDH in *B. amyloliquefaciens* led to a higher level of NADH pool and a lower level of NAD^+^ pool when compared to the parent strain (Figures S3A and S3B). Correspondingly, it was found to increase the ratio of NADH/NAD ^+^ (Figure S3C).

**Figure 3:**
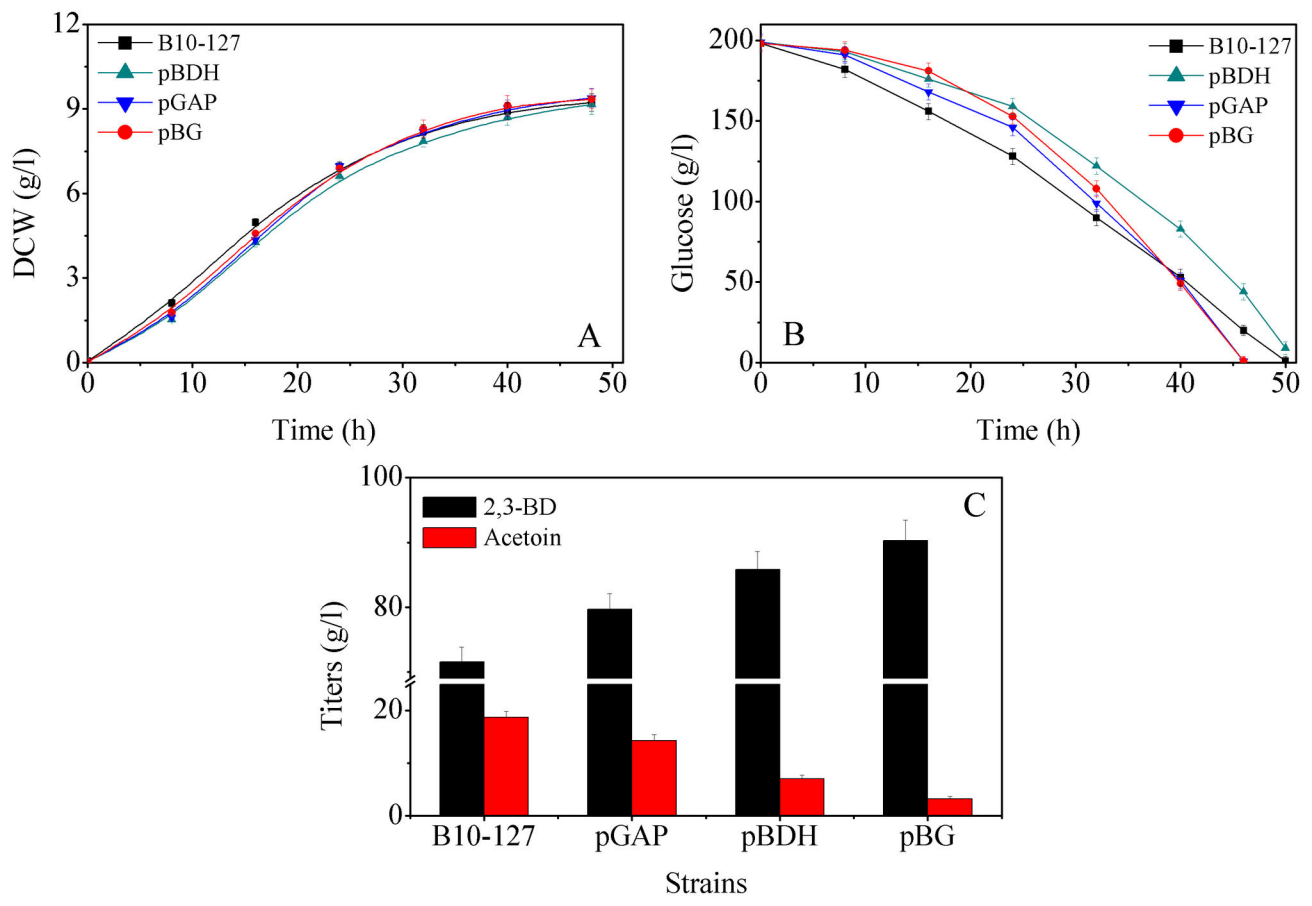
Effects of over-expressing *gapA* and *bdh* on cell growth and 2,3-BD production.

The effects of *gapA* gene over-expression on cell growth and 2,3-BD production were also observed. As shown in Figure 3A, over-production of GAPDH in *B. amyloliquefaciens* did not inhibit cell growth. However, as shown in Figure 3B, the glucose consumption rate of the pGAP strain was slower than that of the parent strain during the growth phase. Surprisingly, marked differences between the parent and mutant strains appeared during the stationary phase. The fermentation rate of the engineered pGAP strain was markedly increased, thereby reducing the fermentation time. As expected, the average glucose consumption rate was enhanced in the pGAP strain (Figure 3B), indicating that over-production of GAPDH increased the rate of glycolysis. Also, relative to the wild type strain (B10-127), the maximum 2,3-BD titer was increased by 11.3%, while that of acetoin was decreased by 23.5% (Figure 3C).

### Over-expression of *bdh* gene and its effects on glucose fermentation to 2,3-BD in *B. amyloliquefaciens*

2,3-BD dehydrogenase (BDH, encoded by *bdh*) catalyzes the conversion of acetoin to 2,3-BD, with concomitant oxidation of NADH to NAD^+^. BDH was also cloned from *B. amyloliquefaciens* B10-127 and over-expressed in the pBDH strain, which harbored the pMA5-*bdh* plasmid. First, the plasmid genetic rate was found to be stable around 96%, indicating that pMA5-*bdh* was stably expressed in pBDH. As shown in Figure S1, BDH was successfully over-expressed in the pBDH strain. Furthermore, as shown in Figure S2B, the specific activity of BDH was 2.21-fold higher than in the wild type strain. And at 24^th^ h, it was about 3.74-fold higher than in the parental strain. And then their activities reached the stationary phase. Again, this result indicates that increasing the gene dosage improves the quantity and performance of BDH.

Over-production of BDH in *B. amyloliquefaciens* was expected to enhance the 2,3-BD branch competing for NADH. As shown in Figure S3, over-production of BDH in *B. amyloliquefaciens* led to a lower level of NADH pool and a higher level of NAD^+^ pool when compared to the parent strain. Correspondingly, it was found to decrease the ratio of NADH/NAD ^+^ (Figure S3C).

Next, the effects of *bdh* over-expression on cell growth and 2,3-BD production were observed. As shown in Figure 3A, cell growth of the pBDH strain was uninhibited by BDH over-production. Relative to the parent strain (B10-127), the glucose consumption rate was slightly reduced in the pBDH strain, implying a longer fermentation time (Figure 3B). However, BDH over-production in *B. amyloliquefaciens* increased the maximum concentration of 2,3-BD by 19.8%, and decreased the acetoin titer by 62.8% (Figure 3C).

### Co-overproduction of genes *bdh* and *gapA* and its effect on glucose fermentation to 2,3-BD in *B. amyloliquefaciens*

The *bdh* and *gapA* genes were cloned from *B. amyloliquefaciens* and over-expressed in the pBG strain. The plasmid genetic rate of the resulting pBG recombinant remained stable at about 95%, indicating that the pMA5-*bdh*-HapII-*gapA* was stably expressed in the pBG. As shown in Figure S1, GAPDH and BDH were successfully co-overexpressed in the pBG strain. The specific activities of BDH and GAPDH in the B10-127 and pBG strains were also determined, as described in Materials and Methods. As shown in Figure S2, at 12^th^ h, the specific activities of GAPDH and BDH in the pBG strain were respectively 1.16-fold and 2.18-fold higher than in the B10-127 strain. And at 24^th^ h, it was about 3.09-fold and 3.71-fold higher than in the parental strain. Again, this result indicates that increasing the genes dosage improve the quantity and performance of GAPDH and BDH.

Intracellular concentrations of NADH and NAD^+^ were fluctuated during various stages of the batch fermentation, and NADH and NAD^+^ levels decreased during growth phases and remained constant during non-growth phases (Figure S3). However, in both parental and engineered strains, the intracellular NADH concentration continuously varied and had no difference during the glucose fermentation, possibly because introducing extra copies of GAPDH/BDH enzymes into *B. amyloliquefaciens* accelerated the NADH/NAD ^+^ regeneration rate without influencing the NAD^+^/NADH levels.

The effects of *bdh* and *gapA* co-overexpression on *B. amyloliquefaciens* cell growth were investigated in batch cultivations of B10-127 and pBG strains. As shown in Figure 3A, the cell growth of both strains was divisible into two stages: fast growth during the first 32 h, followed by a decrease. The growth rates of both strains were similar, suggesting that the co-overexpression of GAPDH and BDH in *B. amyloliquefaciens* did not inhibit cell growth.

As shown in Figure 3B, the pBG strain consumes glucose more slowly than the parent strain during the growth phase. However, the fermentation rate of the engineered pGPD strain significantly increased in the stationary phase, with consequent reduction in fermentation time. In addition, co-overproduction of BDH and GAPDH in *B. amyloliquefaciens* increased the maximum 2,3-BD concentration by 26.1%, while decreasing the acetoin titer by 83.0% (see Figure 3C).

### Effects of over-expression of *bdh* and *gapA* genes on metabolic flux redistributions

**Figure 4:**
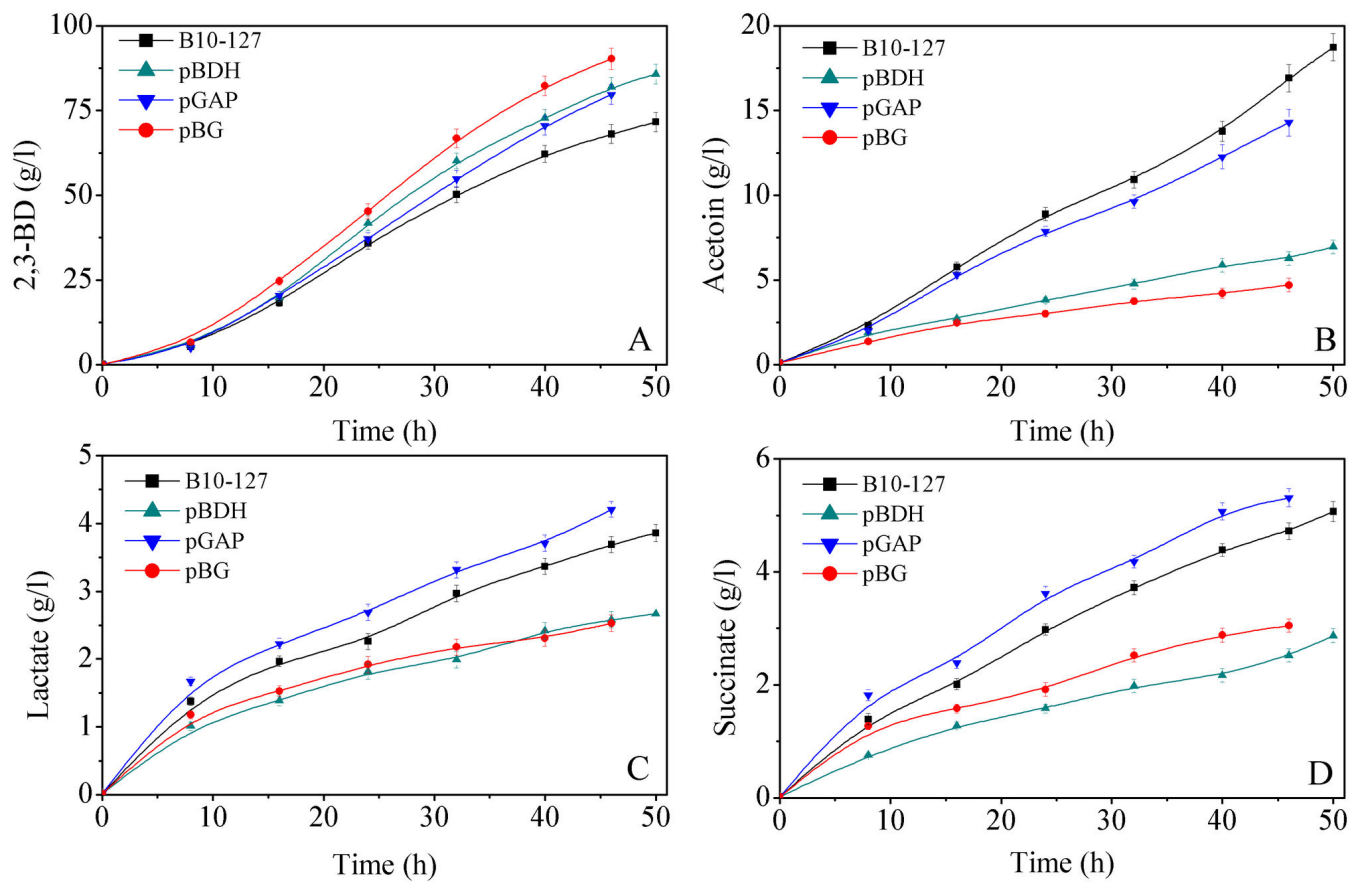
Effects of over-expressing *gapA* and *bdh* on product formation.

In the aerobic glucose metabolism of *B. amyloliquefaciens*, 2,3-BD plays a major role in oxidizing NADH. To secure NADH, it must compete with the end products of pyruvate-deriving pathways (such as lactic acid, succinic acid, and ethanol). Thus, this study characterized the metabolic flexibilities of *B. amyloliquefaciens* in response to over-expression of the *bdh* and *gapA* genes. For this purpose, the concentrations of major metabolites of all strains (B10-127, pBDH, pGAP, pBG) were determined (titers of 2,3-BD, acetoin, succinate, lactate). The results are shown in Figure 4.

The production rates of 2,3-BD, acetoin, lactate and succinate were compared between the mutant (pBDH, pGAP, pBG) and parent strains. Throughout the fermentation process, mutant strains produced 2,3-BD at higher rates (Figure 4A), and yielded acetoin at lower rates (Figure 4B) than the parental strain. The pGAP strain, producing excess GAPDH, showed enhanced rates of 2,3-BD, lactate and succinate production (Figure 4C and 4D). This result indicates that the NADH-dependent pathways (from pyruvate to 2,3-BD and from pyruvate to succinate and lactate) are all improved by over-expressing GAPDH in *B. amyloliquefaciens*. However, over-production of BDH in this strain enhanced 2,3-BD production, while lactate and succinate production were suppressed. This observation demonstrates that 2,3-BD production from glucose is enhanced in the NADH-dependent pathways, while succinate and lactate production from pyruvate are inhibited.

The metabolic flexibilities of *B. amyloliquefaciens* responding to co-overproduction of BDH and GAPDH, are presented in Table 2. The 2,3-BD molar yield was 26.1% higher in pBG than in the B10-127 strain. Compared with the parent strain, the molar yields of unwanted by-products were significantly lower in the mutant strain (82.9%, 33.3% and 39.5% for acetoin, lactate and succinate, respectively). This result indicates that glucose fluxes are redistributed by BDH and GAPDH co-overproduction in *B. amyloliquefaciens*.

**Table 2:** Effects of co-overexpressing BDH and GAPDH on glucose metabolic flux distribution in *B. amyloliquefaciens* (unit: mol/mol glucose).

	**Flux to**				
**Strains**	**2,3-BD**	**Acetoin**	**Lactate**	**Succinate**	**Biomass**
B10-127	0.716	0.187	0.039	0.038	0.056
pBG	0.903	0.032	0.026	0.023	0.058
Fluxes redistributed	26.1%	-82.9%	-33.3%	-39.5%	3.57%

### Fed-batch production of 2,3-BD

**Figure 5:**
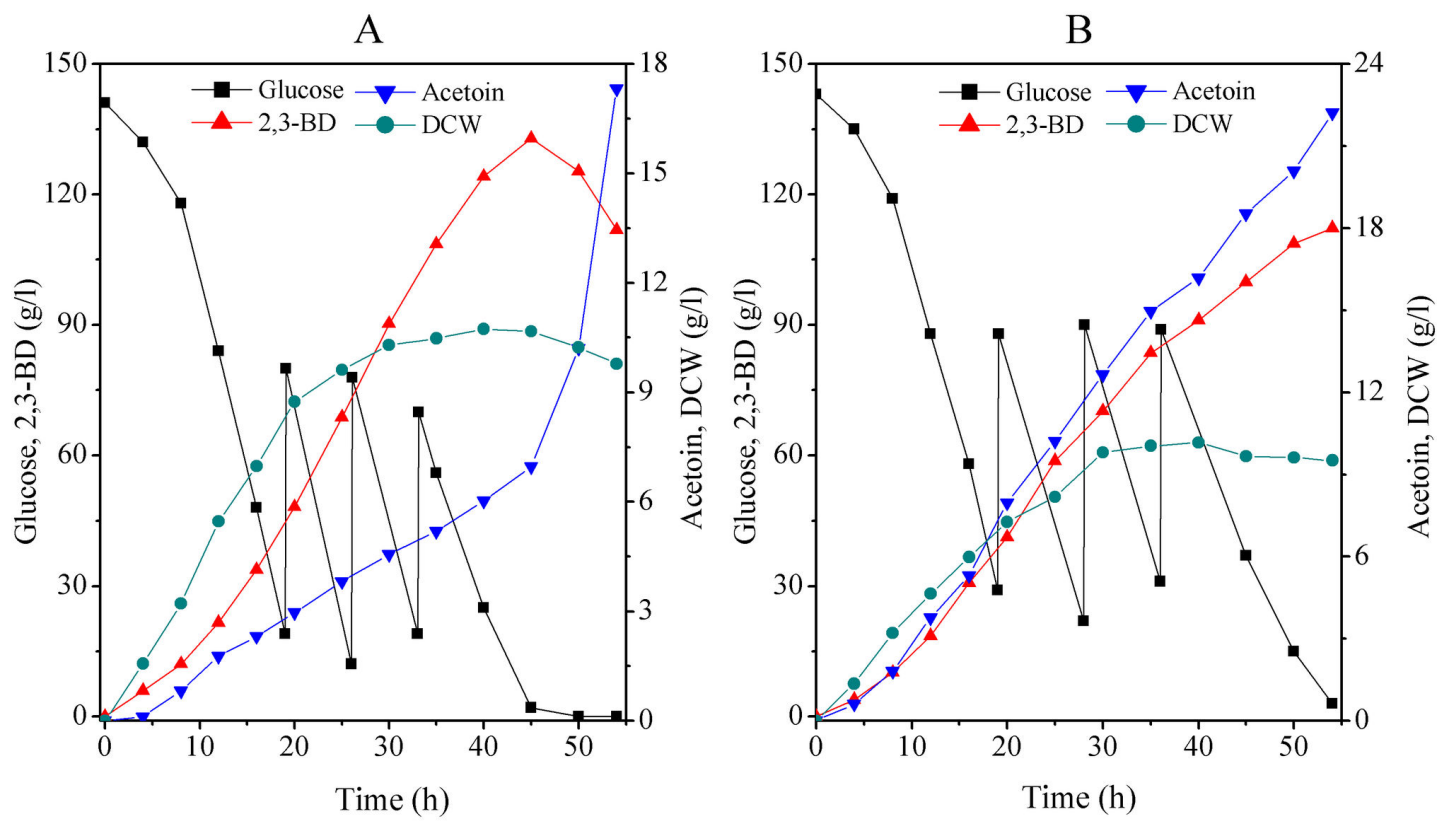
Fed-batch fermentation of 2,3-BD in a 5-l bioreactor. ((A) inoculated by the engineered strain pBG; (B) inoculated with the parental strain).

Fed-batch production is a widely-applied industrial process. However, because batch fermentation cultures are inhibited by high substrate concentration, the target 2,3-BD concentration and productivity are not reached in these cultures. Substrate limitation and inhibition can be avoided in fed-batch cultures by retaining the substrate concentration in the reactor below toxic levels [26]. Fed-batch culture of the pBG strain and parental strain were conducted in separate agitated bioreactors under the following conditions: initial pH 6.5, temperature 37 °C, agitation speed 350 rpm and aeration rate 0.33 vvm. Glucose solution (800 g/l) was pumped into the bioreactor by a pulse feeding strategy. Figure 5 plots the changes in concentrations of residual glucose, cell mass, acetoin, and 2,3-BD during the fed-batch fermentation. As shown in Figure 5A, batch cultures of the pBG strain produced 2,3-BD at a maximum rate of 132.9 g/l. This rate was achieved after 45 h; the corresponding productivity was 2.95 g/l h, while the acetoin concentration was only 6.98 g/l. By contrast, the performance of the parental strain in batch culture was unsatisfactory (Figure 5B), yielding a maximum 2,3-BD concentration of 112.3 g/l after 54 h. More detrimentally, acetoin had accumulated to 22.1 g/l. The above results indicate that co-overexpression of *bdh* and *gapA* genes is an effective means of improving 2,3-BD production while inhibiting the accumulation of unwanted byproducts.

## Discussion

The potential for large-scale production of 2,3-BD by *B. amyloliquefaciens* has been previously demonstrated [12,13], but fermentation was accompanied by undesirably large production of acetoin, lactate and succinate, while the 2,3-BD yield remained too low for commercial viability. Reducing these by-products is beneficial to 2,3-BD production, because they compete with 2,3-BD for pyruvate as a metabolic intermediate and NADH as a cofactor. Therefore, increasing 2,3-BD production by *B. amyloliquefaciens* is an economically valuable goal.

Assimilation of glucose to generate 2,3-BD constitutes an oxido-reduction-associated process (Figure 1). In cofactor-dependent production systems, cofactor availability and the proportion of cofactor in the active form may largely determine the overall process yield [27]. One function of the 2,3-BD pathway is to regenerate the excess reducing power associated with glycolysis [5]. Thus, the 2,3-BD pathway participates in regulating the intracellular NADH/NAD ^+^ ratio. As mentioned earlier, intracellular acetoin accumulation is likely governed by the quantities of rate-limiting factor BDH and/or NADH (the latter is preferentially used for 2,3-BD synthesis in this strain).

Glyceraldehyde-3-phosphate dehydrogenase (GAPDH, encoded by *gapA*) is a key enzyme in the glycolytic pathway that catalyzes the oxidative phosphorylation of glyceraldehyde-3-phosphate to 1,3-bisphosphoglycerate. This reaction is coupled to the reduction of oxidized nicotinamide adenine dinucleotide, (NAD^+^) to NADH [28]. Therefore, GAPDH over-expression might enhance 2,3-BD production by increasing the level of available NADH. Inspired by this idea, we initially over-produced NAD^+^-dependent GAPDH in *B. amyloliquefaciens*, which increased not only the 2,3-BD yield, but also lactate and succinate production. If GAPDH over-expression indeed increases the available NADH, all of the NADH-dependent pathways, (2,3-BD, lactate and succinate pathways) would benefit from the enhancement.

In the presence of BDH, one mole of acetoin is converted to an equimolar 2,3-BD, with concomitant oxidation of NADH to NAD^+^ [29]. Subsequently, NADH-dependent BDH was over-produced in *B. amyloliquefaciens*, resulting in high production of 2,3-BD with suppressed acetoin, lactic acid and succinic acid. Two reasons may account for this phenomenon. First, over-production of BDH might promote the conversion of acetoin into 2,3-BD. Second, in the glucose metabolism of *B. amyloliquefaciens*, the 2,3-BD branch is primarily responsible for oxidizing NADH. When NADH-dependent BDH is over-expressed, the 2,3-BD branch gains a competitive advantage over the end products of pyruvate-deriving pathways (such as lactic acid and succinic acid), through the enhanced availability of NADH. Heterologous expression of 2,3-BD genes in engineered *E. coli* has been reported under fermentative conditions [30]. The engineered strain contained the *alsS* and *alsD* genes from *B. subtilis* and the *bdh* gene (encoding BDH) from *K. pneumoniae*. The mutant *E. coli* strain, implementing the 2,3-BD producing pathway under aerobic conditions, demonstrated higher glucose consumption and faster 2,3-BD production rate than the wild-type control [30]. Furthermore, Biswas et al [10] developed a *B. subtilis* strain for enhanced 2,3-BD production in the early log phase of the growth cycle. By placing the *bdh* gene under the control of the PalsSD promoter in the AlsSD operon, they improved the 2,3-BD production up to 6.1 g/l. Acetoin accumulation is important in the wine industry, since acetoin exerts a negative sensory impact on wine. Ehsani et al. [31] found that, by over-producing BDH, 85 to 90% of the accumulated acetoin could be redirected into 2,3-BD, a compound with neutral sensory characteristics. The above results demonstrate that over-expression of BDH might enhance 2,3-BD production. At the same time, the 2,3-BD yields of model strains (such as *E. coli* and *B. subtilis*) remain prohibitively low for commercial production. Therefore, in an initial trial, NAD^+^-dependent GAPDH and NADH-dependent BDH were co-overexpressed in *B. amyloliquefaciens*. Surprisingly, strain pBG (harboring both *bdh* and *gapA* genes) produced more 2,3-BD and accumulated less succinate and lactate than the other strains (B10-127, pGAP, pBDH).

To improve 2,3-BD production and reduce the unwanted by-products, some researchers have deleted the undesired genes. Jung et al. [32] reduced lactate production by deleting the gene encoding lactate dehydrogenase from *E. aerogenes*. As expected, 2,3-BD production was much higher in the deletion mutant than in its parental strain. Shen et al. [33] deleted the genes responsible for acetate production (*ack*A and *pox*B) to achieve reduced acetate formation and enhanced 2,3-BD production. Han et al. [34] constructed low-acid producing *K. oxytoca* NBRF4 by chemical mutation and screening against NaBr, NaBrO3 and fluoroacetate. This mutant showed moderate reduction in acetate and succinate production, yielding 46% fewer total acids than the wild-type strain. The concentration and productivity of 2,3-BD was also 9-21% higher in the NBRF4 mutant. In the current study, we found that co-overexpression of *bdh* and *gapA* genes effectively improved the 2,3-BD production while inhibiting acetoin, lactate and succinate accumulation.

Higher 2,3-BD titers could be achieved by an effective fermentation strategy. Therefore, we amplified the productivity and concentration of 2,3-BD by batch-fed fermentation. The maximum 2,3-BD concentration obtained in batch culture was 132.9 g/l after 45 h. The corresponding 2,3-BD productivity was 2.95 g/l·h. To our knowledge, we report the highest 2,3-BD production levels thus far obtained from fermentation by a *Bacillus* sp (an excellent candidate for industrial-scale microbial fermentation of 2,3-BD). The yield and productivity of strain pBG might be comparable to the most efficient but toxic 2,3-BD producers *K. pneumoniae*, *K. oxytoca* and *E. aerogenes* [4].

## Conclusion

Over-production of NAD^+^-dependent GAPDH improved the 2,3-BD productivity but also increased production of the undesirable by-products lactate and succinate. On the other hand, over-production of NADH-dependent BDH increased the 2,3-BD production while repressing lactate and succinate production, at the expense of fermentation time. However, co-overexpression of the two key enzymes BDH and GAPDH in the NADH-dependent pathways of *B. amyloliquefaciens* yielded both high 2,3-BD titer and fewer by-products (acetoin, lactate and succinate). These results indicate that co-overexpression of *bdh* and *gapA* genes can effectively improve 2,3-BD production while inhibiting the accumulation of unwanted by-products.

## Supporting Information

Figure S1 **SDS-PAGE analysis of BDH and GAPDH expression levels.**
(TIF)

Figure S2 **Time profiles of the specific activities of GAPDH and BDH.**
(TIF)

Figure S3 **Effects of over-expressing the **NADH**/**NAD** ^+^ regeneration system in *Bacillus amyloliquefaciens* on the concentrations of intracellular NADH, NAD^+^ and **NADH**/**NAD** ^+^ ratio.**
(TIF)
