# Supplementary figures and images for: Improved Production of 2,3-Butanediol in Bacillus amyloliquefaciens by Over-Expression of Glyceraldehyde-3-Phosphate Dehydrogenase and 2,3-butanediol Dehydrogenase
Source: PLoS One. 2013 Oct 2;8(10):e76149. doi: 10.1371/journal.pone.0076149 (PMC3788785; doi:10.1371/journal.pone.0076149)

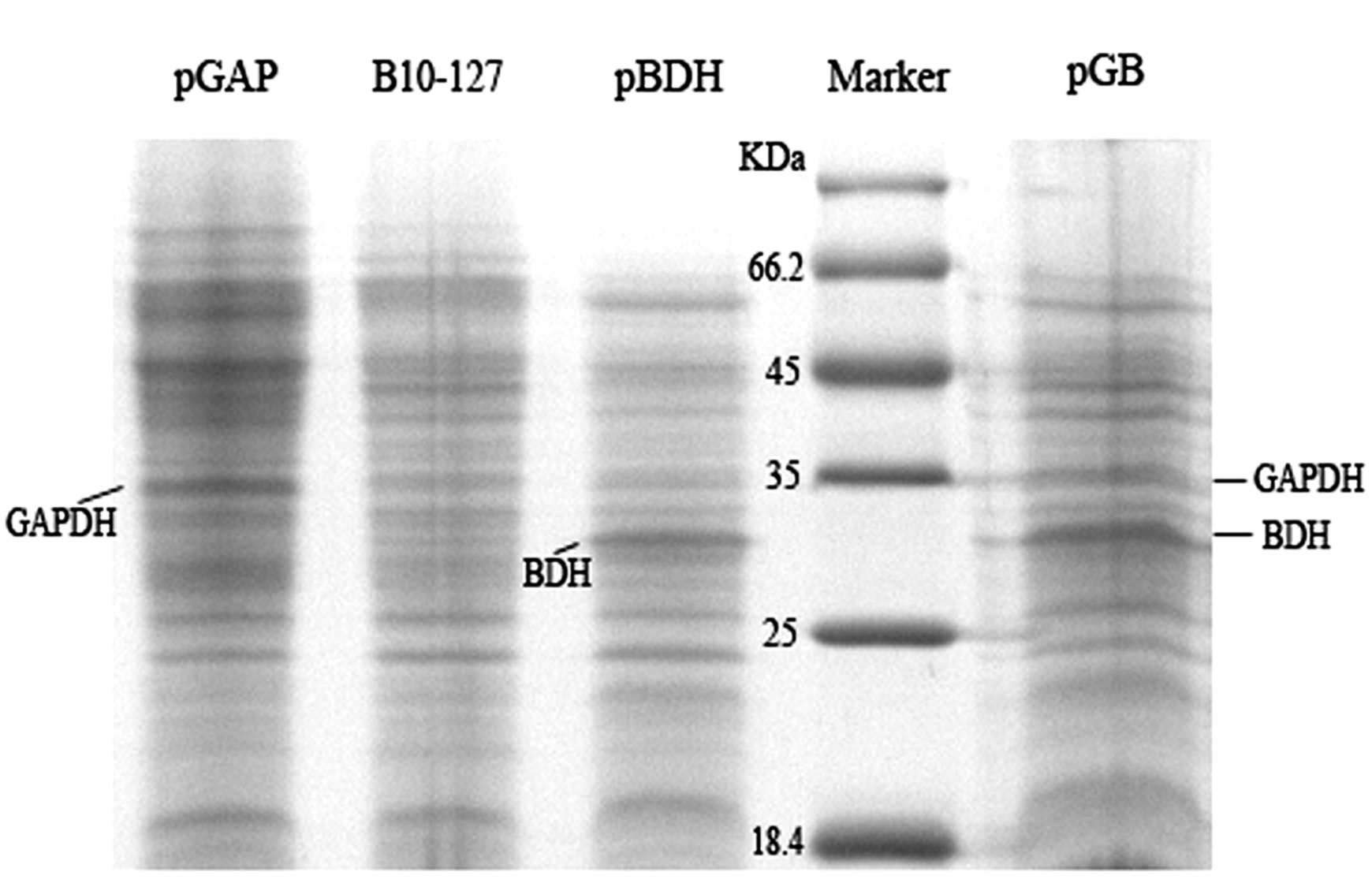

Supplement: Figure S1 — SDS-PAGE analysis of BDH and GAPDH expression levels. (TIF) [file pone.0076149.s001.tif]

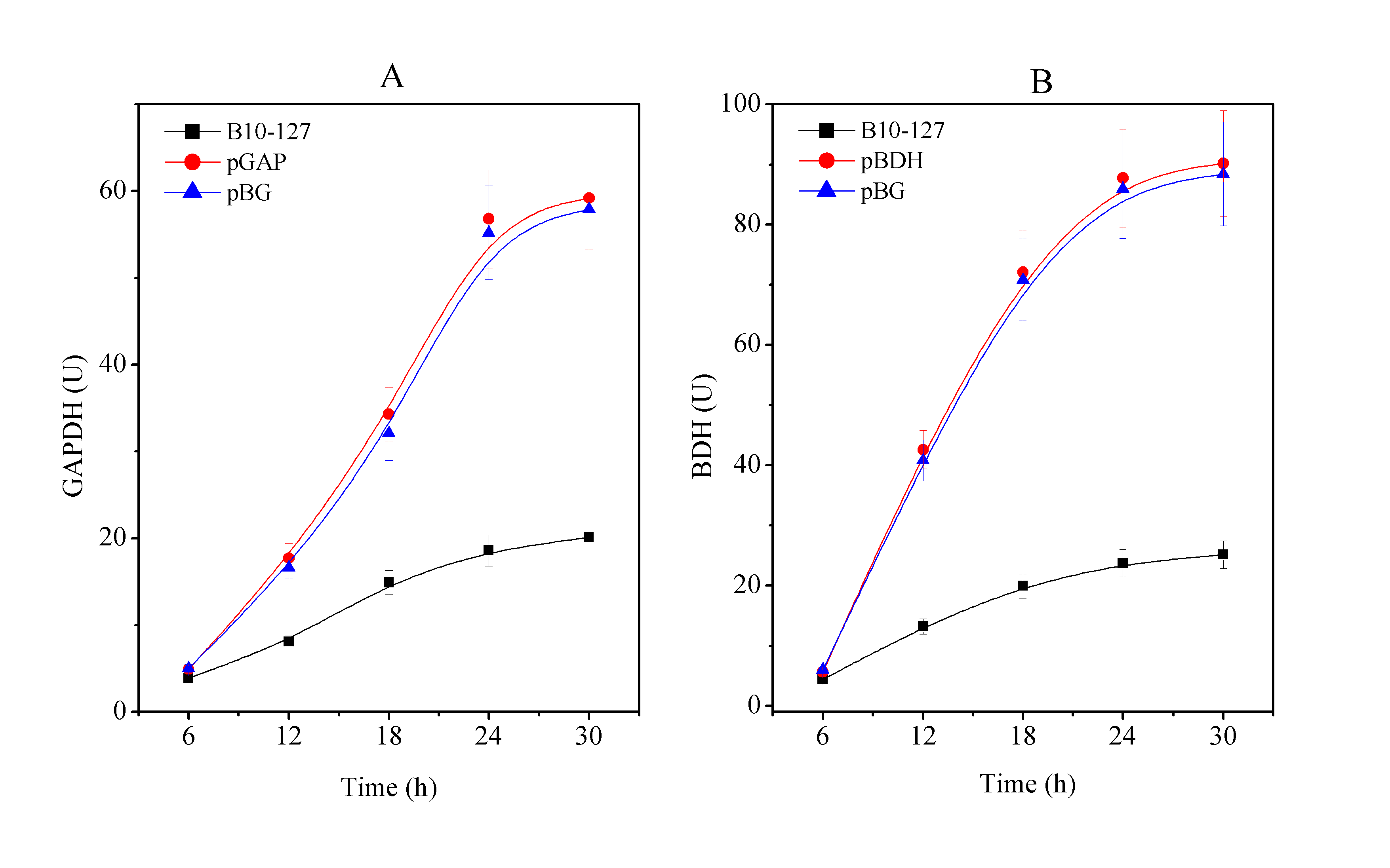

Supplement: Figure S2 — Time profiles of the specific activities of GAPDH and BDH. (TIF) [file pone.0076149.s002.tif]

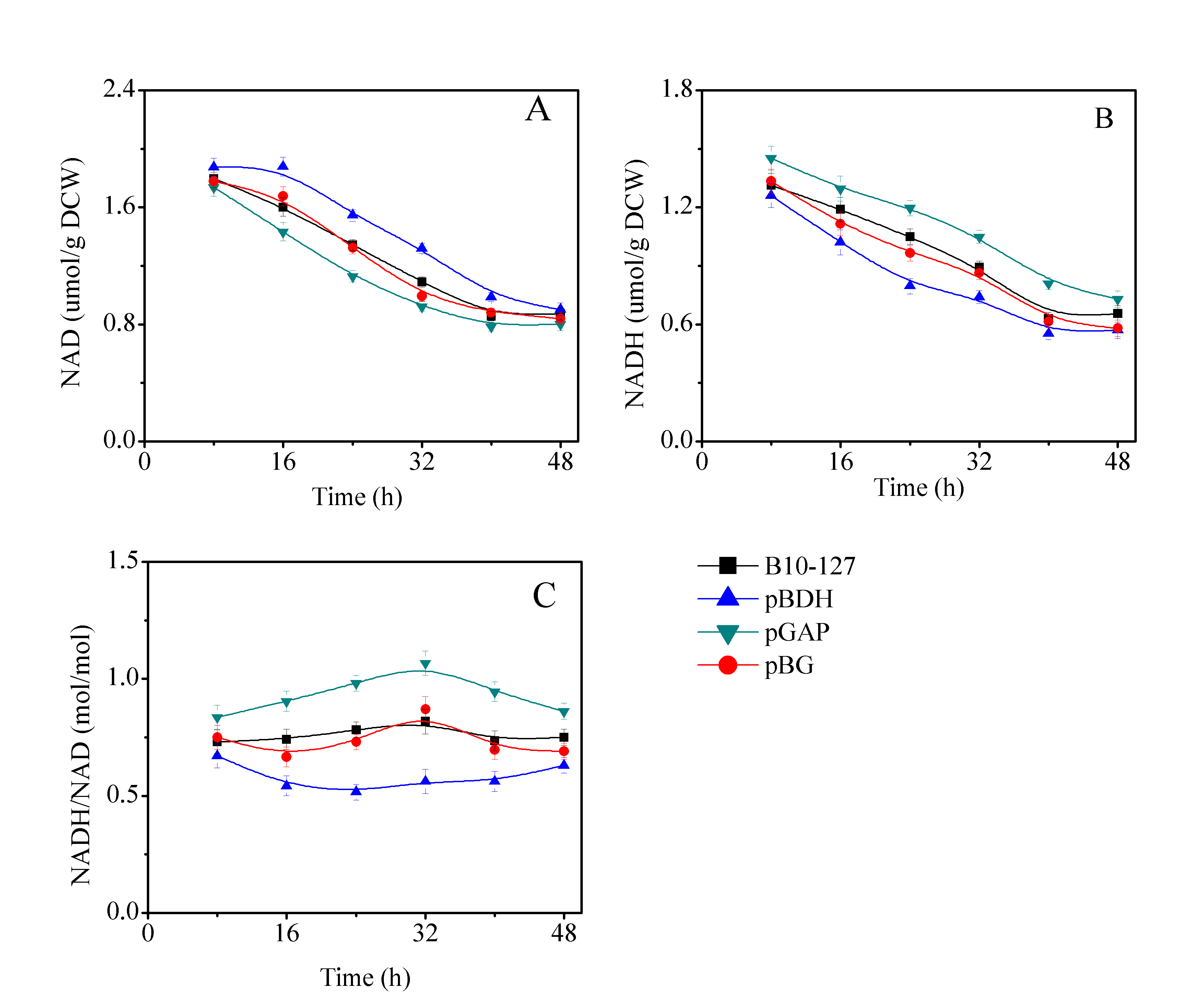

Supplement: Figure S3 — Effects of over-expressing the NADH/NAD + regeneration system in Bacillus amyloliquefaciens on the concentrations of intracellular NADH, NAD+ and NADH/NAD + ratio. (TIF) [file pone.0076149.s003.tif]
